# Supplementary material for: Coherent structural trapping through wave packet dispersion during photoinduced spin state switching
Source: Nat Commun. 2017 May 24;8:15342. doi: 10.1038/ncomms15342 (PMC5458100; doi:10.1038/ncomms15342)
Supplement: Supplementary Information — Supplementary Figures, Supplementary Notes, Supplementary Table and Supplementary References [file ncomms15342-s1.pdf]

## Supplementary Note 1: Global fit of the XANES data at different energies

Fig. 2 shows the time-dependent change of XANES intensity $\Delta I^E(t)$ for different energy $E$, normalized to the XANES intensity in the LS ($I_{off}$) ground state. It is analyzed as a superposition of the signal from the MCLT and HS states, schematically represented in Supplementary Fig. 1:

$$\Delta I^E(t)/I_{off} = S_{MLCT}(t) + S^E_{HS}(t)$$

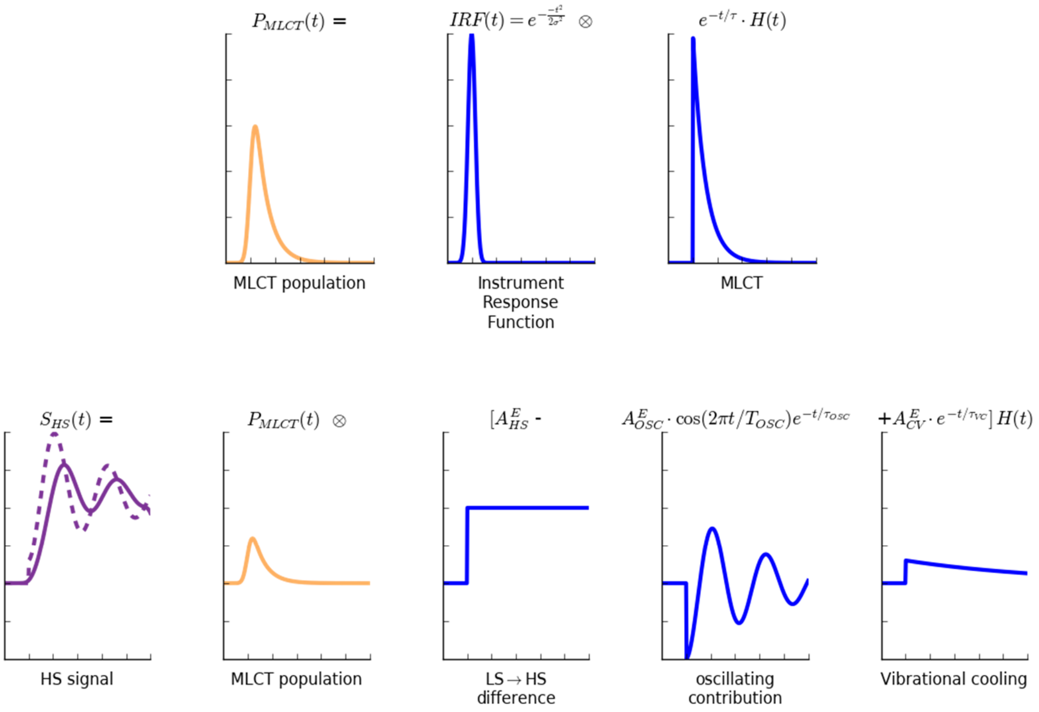


**Supplementary Figure 1.** intuitive construction of the phenomenological model. The top panels refer to the MLCT population that is obtained by convoluting an exponential decay with a Gaussian function representing the Instrument Response Function (IRF). The MLCT population is then used as “source” of HS molecules by convolution with the HS signal. The dashed line represent the HS contribution before convolution. Reduced oscillation amplitudes and a shift in the time axis are the most visible features.

At a given energy $E$, the contribution to the XANES signal of the MLCT state which decays with a time constant is given by: $S^E_{MLCT}(t) = A^E_{MLCT} \times P_{MLCT}(t)$

where $A^E_{MLCT}$ is the amplitude of the MLCT, $P_{MLCT}(t) = IRF(t) \otimes [\, exp(-t/\tau_{MLCT}) \cdot H(t)]$, $\otimes$ is the convolution operator, $IRF(t)$ is the instrument response function - assumed to be a Gaussian $IRF(t) = \frac{1}{\sqrt{2\pi\sigma}} exp(-t^2/2\sigma^2_{IRF})$- and $H(t)$ the Heaviside function.

The signal from the HS state is described as:

$s^E_{HS}(t) = [\, A^E_{HS} - A^E_{OSC} cos(2\pi t/T_{OSC})\, exp(-t/\tau_{OSC}) + A^E_{VC} exp(-t/\tau_{VC})\,] \cdot H(t)$,

where $A^E_{HS}$ is the signal due to the final HS state after cooling, $A^E_{OSC}$ is the amplitude of oscillation, $\tau_{OSC}$ is the damping of the oscillation of period $T_{OSC}$, $A^E_{VC}$and $\tau_{VC}$are the amplitude of the signal and the timescale of the vibrational cooling. Since the MLCT state is the source of molecules in the HS state, $s^E_{HS}(t)$is convoluted with the $P_{MLCT}(t)$ to give rise to the part of the signal due to the HS state $S^E_{HS}(t) = P_{MLCT}(t) \otimes s^E_{HS}(t)$. Please note that for a time independent $s^E_{HS}(t) = A^E_{HS}$, the signal due to the HS state reduces to:

$$S^E_{HS}(t) = P_{MLCT}(t) \otimes s^E_{HS}(t) = P_{MLCT}(t) \otimes [A^E_{HS}\, H(t)] = A^E_{HS}\, P_{HS}(t).$$

All parameters with an *E* superscript are allowed to vary independently. Due to uncertainty in the time zero stability between the different energies, time traces at each energy are allowed to be shifted in time by $t^E_0$ (limited to +/- 30 fs). The resulting values of $A^E_{MLCT}$ plotted in Fig. 1a identify the state as MLCT due to the similarities with the calculated MLCT signal considered as resulting from a +1eV shift because the formal oxidation state is $Fe^{3+}$ in the MLCT suggested by previous XANES studies on similar compounds [1,2].

The physical parameters describing the mechanism with the decay from MLCT to HS accompanied with coherent HS oscillation and vibrational cooling were obtained by a global fitting of the XANES data at different energies in Fig. 2:

- the time decay of the MLCT $\tau_{MLCT}$=120(10) fs,
- the oscillation period $T_{OSC}$=265(10) fs (corresponding to 126(3) $cm^{-1}$),
- the oscillation damping timescale$\tau_{OSC}$ =330(10) fs,
- the vibrational cooling decay$\tau_{VC}$=1.6(0.1) ps.
- the RMS width of the IRF ($\sigma_{IRF}$) = 25(5) fs.

The energy dependent parameters along with the uncertainty estimates are reported in Supplementary Table 1.

In Fig. 2b, the $r_1$curve is traced using the timescale parameters found by the global fit:

$$r_1(t) = 0.2 - 0.2\,[cos(2\pi t/T_{OSC})exp(-t/\tau_{OSC})]$$

$r_1(t)$ represents the average trajectory if all molecules would reach the HS state at t=0 (i.e. if $\tau_{MLCT} = 0$).

| Photon energy (eV) | $t_0$ (ps) | $A^{MLCT}$ | $A^{HS}$ | $A^{VC}$ | $A^{OSC}$ |
|---|---|---|---|---|---|
| 7113.5 | 0.018 ± 0.002 | -0.045 ± 0.001 | -0.038 ± 0.004 | 0.0 (Fixed) | 0.0 (Fixed) |
| 7121.5 | -0.037 ± 0.003 | -0.24 ± 0.03 | 0.476 ± 0.004 | 0.094 ± 0.007 | 0.355 ± 0.005 |
| 7132.5 | -0.01 ± 0.00 | 0.054 ± 0.002 | -0.015 ± 0.001 | -0.01 ± 0.001 | 0.111 ± 0.002 |
| 7145 | 0.003 ± 0.001 | 0.016 ± 0.002 | -0.171 ± 0.002 | 0.005 ± 0.001 | 0.131 ± 0.002 |
| 7155 | 0.005 ± 0.002 | 0.009 ± 0.001 | -0.023 ± 0.002 | 0.048 ± 0.003 | 0.078 ± 0.001 |
| 7156 | 0.006 ± 0.002 | 0.006 ± 0.002 | 0.013 ± 0.002 | 0.041 ± 0.003 | 0.113 ± 0.002 |
| 7164 | -0.008 ± 0.002 | -0.013 ± 0.001 | 0.178 ± 0.001 | -0.021 ± 0.001 | 0.124 ± 0.002 |

**Supplementary Table 1**: Energy dependent parameters obtained from the global fit.

## Supplementary Note 2: XANES signal simulation

The XAS calculations shown in Supplementary Fig. 2a were performed using the MXAN code [3]. It uses the so-called full multiple scattering approach, avoiding any a priori selection of the relevant multiple scattering paths, together with the muffin-tin (MT) approximation for the shape of the atomic potentials [4]. The Coulomb and exchange parts of the total cluster potential were calculated using the total charge density, which was approximated using a superposition of spherically symmetric self-consistent (SCF) atomic charge densities. These atomic charge densities are generated by SCF relativistic Dirac-Fock atomic code that automatically runs in MXAN for all atoms in the Periodic Table. The use of the MT approximation forces the introduction of two free-parameters, the MT radii of the atomic spheres and the interstitial potential that must be optimized in the calculation to get a good agreement between theory and experiment. Such refinement is used to mimic both the SCF potential of the full cluster and compensate for the MT approximation. This is necessary because SCF and non-MT corrections both modify the atomic t-matrices which likewise depend on the muffin-tin radii and the interstitial potential. It points to the possibility of mimicking the effects of the non-MT correction and a full SCF potential by judicious optimization of the radii and the interstitial potential. This theoretical consideration is the basis of the potential optimization procedure normally applied in the MXAN code[3]. Here we have optimized the non-structural parameters by fitting, in terms of non-structural parameters and the Fe-N distance, the low-spin ground-state experimental data coming from LCLS in the energy range from the edge to 60 eV above, obtaining a very good agreement between experimental data and theoretical calculation and a good structural reconstruction with $r$= 2.05±0.04Å. The non-structural parameters derived after the fitting procedure are kept fixed to calculate the series of spectra with a the Fe-N distance ranging from 1.6 Å to 3 Å, with a step of 0.05 Å.

A chemical shift of -1.5 eV has been used for the calculation of the signal for the high spin state. Supplementary Fig. 3 compares the measured XANES signal and its simulation considering the evolution of the distance distributions $g(r,t)$ mentioned above for the ensemble motion model or the evolution of average distance $\bar{r}(t)$.

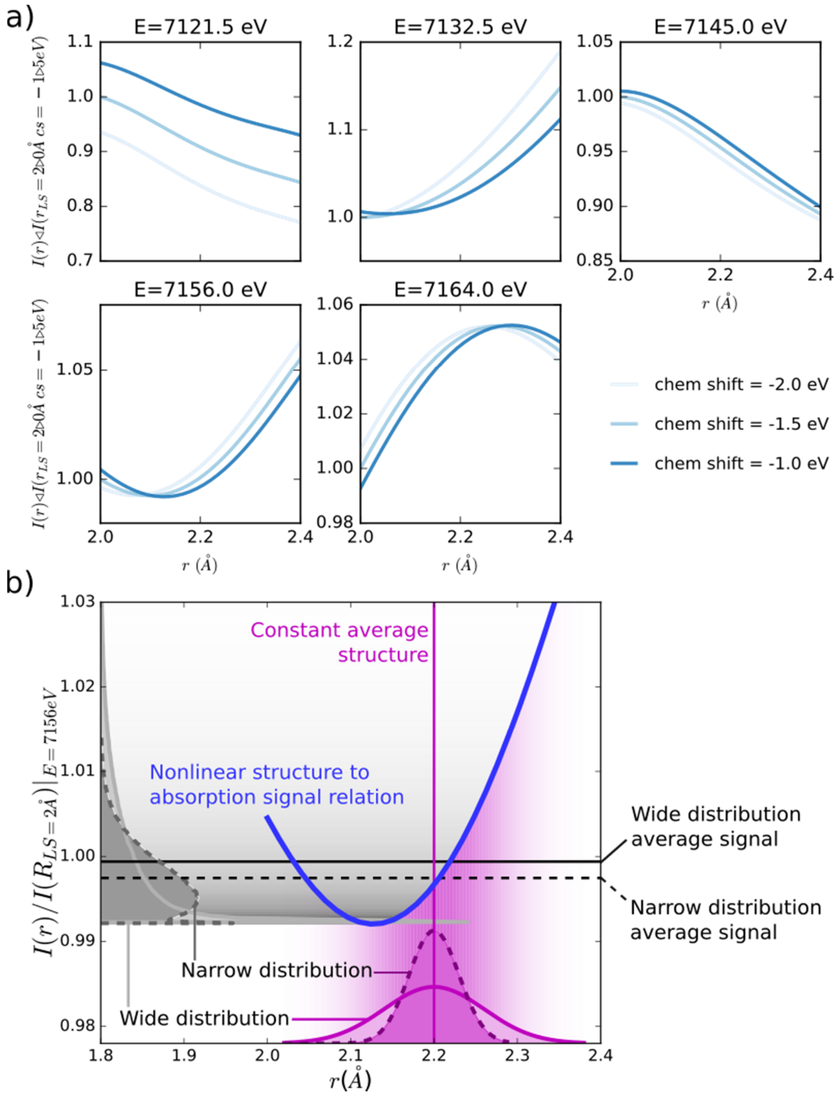


**Supplementary Figure 2**. (a) Dependence of the signal using the multiple scattering calculations as function of the Iron-Nitrogen distance (*r*) for different chemical shift (*cs*). Curves are normalized for the intensity calculated for *r=2 Å* and *cs=-1.5 eV*; At certain energies like 7145 eV the changes are predominantly linear with *r* and show little sensitivity to the chemical shift. Conversely, 7156 eV shows a significant non-linear dependence regardless of the chemical shift. (b) Exaggerated visualisation of influence of the nonlinear dependency of absorption signal (ordinate) to structural coordinate (abscissa): A normal distribution of structures gives rise to a non-symmetric signal distribution (see labels in plot). The average signal, the observed magnitude in the experiment, depends on the distribution width of structures.

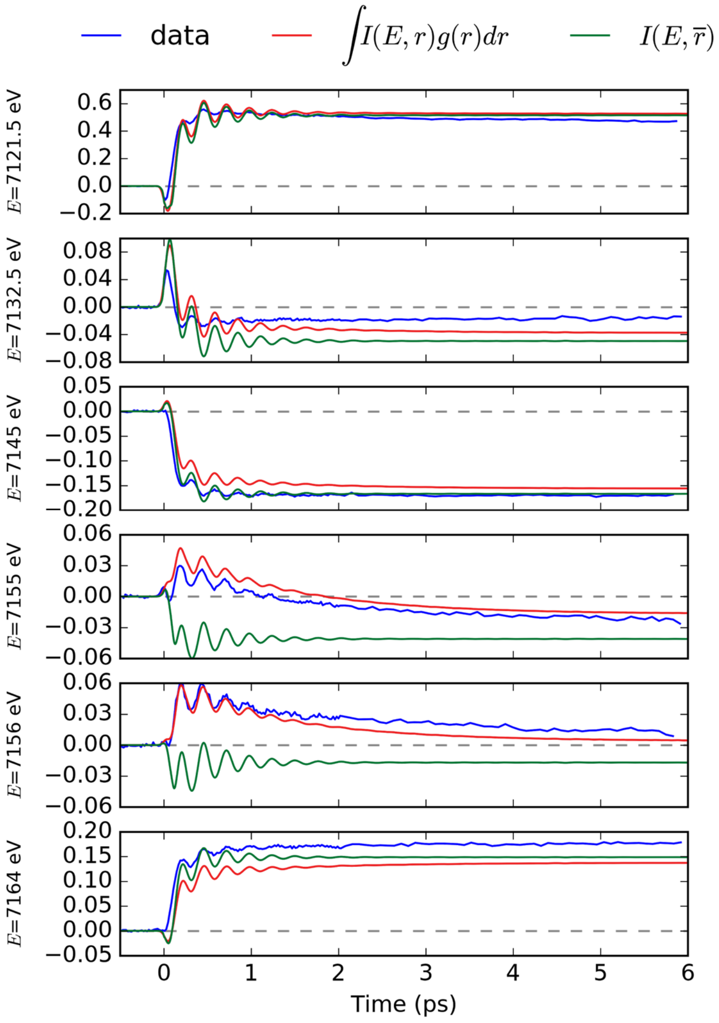


**Supplementary Figure 3.** Expected signals calculated using the distance distributions $g(r,t)$ (red) of an ensemble motion model (red curves) or the average distance $\bar{r}(t)$ change (green curves). The experimental data (blue) is shown for comparison.

## Supplementary Note 3: Estimation of the distribution width from the experimental data

We start from the EXAFS equation, approximating it as resulting only from the first coordination shell with 6 N atoms at the same distance *r*: $\chi(k,r) = \frac{6f(k)}{kr^2} ex^{p}(-2k^2\sigma^2) si^{n}(2kr)$, by rewriting $r = r_{HS} + \Delta r$, where $r_{HS}$ is the HS equilibrium distance;

The ratio assuming unchanged thermal factors becomes:

$\Delta I/I = \chi(k,r)/\chi(k,r_{HS}) - 1 = r_{HS}^2/(r_{HS} + \Delta r)^2 sin[2k(r_{HS} + \Delta r)]/sin[2kr_{HS}] - 1.$ This can be expanded around $\Delta r = 0$ and for an interference minimum $2kr_{HS} = 3/2 \cdot \pi$ as

$$\Delta I/I = -2\Delta r/r_{HS} + \Delta r^2(3/r_{HS}^2 - 2k^2) + \Delta r^3(4k^2/r_{HS} - 4/r_{HS}^3) + \Delta r^4(2/3k^4 - 6k^2/r_{HS}^2 + 5/r_{HS}^4) + O(\Delta r^5)$$

Assuming a normal distribution centered around $\Delta r = 0$ , with a width of $\sigma_r$, we obtain

$\Delta I/I = (3/r_{HS}^2 - 2k^2)\sqrt{2\pi}\sigma_r^3 + (2/3k^4 - 6k^2/r_{HS}^2 + 5/r_{HS}^4)\sqrt{2\pi}\sigma_r^3.$ For values corresponding to $E = 7156.5$ eV $\rightarrow k = 3.3$ Å$^{-1}$, and $r_{HS} = 2.2$ Å, the first term dominates over the second one and we obtain the formula linking the measured signal to the distribution width: $\Delta I/I \simeq -44.5\, \sigma_r^3$.

## Supplementary Note 4: Transient molecular distribution model

To further investigate the origin of the 1.6 ps relaxation, a simple dynamical model for the vibrational mode has been developed that, coupled with XANES calculations, aims to qualitatively reproduce the observed signal. The idea behind this model is that the loss of phase (dephasing) with some loss of energy can be distinguished from a pure damping of vibrational energy (loss of average amplitude) by comparing the transient distribution with the data, which broadens with the first process and narrows with the latter. Moreover, the rather long MLCT lifetime compared with the oscillation period causes a significant loss of phase and helps explain the relatively small ensemble averaged oscillation amplitude (with respect to the step amplitude) at simultaneous strongly increasing delocalisation.

Briefly, starting with thermally distributed position and momentum of the coordinate *r*, the system evolves in the LS potential (characterized by a period of ~170 fs). Each “molecule” of the ensemble is then “promoted” from the LS to MLCT at a given time (taken from a normal distribution representing the IRF with an RMS width of 30 fs; the stochastic MLCT to HS transition is modeled by an exponential time distribution of 120 fs lifetime (that matches the intermediate MLCT). Once promoted, the system evolves in the HS potential along the reaction coordinate *r* characterized by in-phase breathing of the rigid bpy ligands through simultaneous stretching of the six Fe-N bonds around *r* ~2.2 Å. To account for dephasing and damping, energy redistributions events (average time

between them of 400 fs) are considered. They transfer part of the energy to a thermal bath and cause a phase loss by inverting the momentum. To link the dephasing events to the loss of energy, it is assumed that for each event a certain fraction of energy is lost (60% in the model shown). Furthermore, a simple thermostat has been introduced by adding a stochastic change of velocity (normally distributed) to the velocity of the particles. The rms width of this normal distribution is chosen to be $\sqrt{2\gamma K_B T}$ where $\gamma$ is a coupling parameter, $K_B$ the Boltzmann constant and $T$ the (absolute) temperature. The value of $\gamma$ has been chosen to keep the distribution width in the LS state to the value found by Daku and Hauser [5]. The calculation is run - with fixed parameters - for a statistical ensemble of $10^6$ "molecules". The program can be obtained by request to the authors.

From this ensemble of trajectories, the time dependent distance distribution, $g(r,t)$, is easily calculated. The results of the calculations are shown as 2D false colour plot in Fig. 3c. The distribution center of mass moves from $r$ = 2.0 to 2.2 Å when moving from t<0 to several picoseconds. Within 1 ps the oscillating component disappears and on longer time scales the distribution narrows due to cooling in the HS potential. Coupling the obtained distributions with XANES simulations (see Methods) that give the signal as function of energy and displacement ($r$), i.e. $I_{calc}(E,r)$, allows the calculation of the expected signal in two different ways, by integrating the expected signal

$$\Delta I_g = \int I_{calc}(E,r)\, g(r,t)\, dr \text{ or } \Delta I_m = I_{calc}(E,\overline{r}) \text{ with } \overline{r}(t) = \int g(r,t) r\, dr$$

In other words, while $\Delta I_m$ takes into account only the average position of the reaction coordinate, $\Delta I_g$ uses the full information contained in the distribution of the reaction coordinate. The calculated XANES changes are normalized to XANES intensity in the LS ($I_{\text{off}}$).

The process is schematically represented in Video 2.

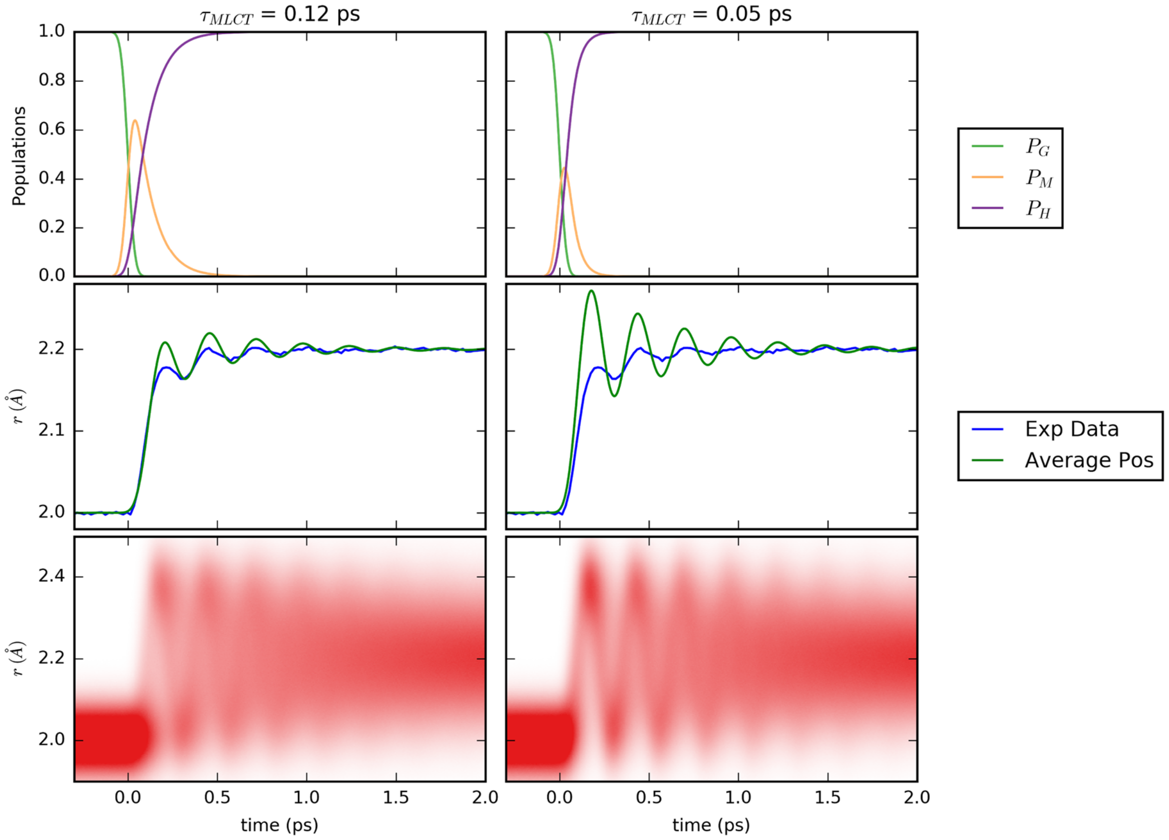


**Supplementary Figure 4**. Population of the LS, MLCT and HS electronic states (top), experimental data at 7145 eV (blue) scaled in $r$ together with the simulated signal (middle), resulting from the time evolution of the distribution of $r$ (bottom). The left part corresponds to the model presented in the manuscript with a single 120 fs lived MLCT intermediate. The right part corresponds to a single 50 fs lived MLCT intermediate.

## Supplementary References


1. Gawelda, W. *et al.* Electronic and molecular structure of photoexcited [Ru(II)(bpy)3]2+ probed by picosecond X-ray absorption spectroscopy. *J. Am. Chem. Soc.* **128,** 5001–9 (2006).
2. Cafun, J.-D. *et al.* Room-Temperature Photoinduced Electron Transfer in a Prussian Blue Analogue under Hydrostatic Pressure. *Angew. Chemie Int. Ed.* **51,** 9146–9148 (2012).
3. Benfatto, M. & Della Longa, S. MXAN: New improvements for potential and structural refinement. in *Journal of Physics: Conference Series* **190,** 12031 (2009).
4. Benfatto, M. & Della Longa, S. Geometrical fitting of experimental XANES spectra by a full multiple-scattering procedure. *J. Synchrotron Radiat.* **8,** 1087–1094 (2001).
5. Lawson Daku, L. M. & Hauser, A. Ab Initio Molecular Dynamics Study of an Aqueous Solution of [Fe(bpy) 3 ](Cl) 2 in the Low-Spin and in the High-Spin States. *J. Phys. Chem. Lett.* **1,** 1830–1835 (2010).
